# Optimization of In Vitro Transcription by Design of Experiment to Achieve High Self-Amplifying RNA Integrity

**DOI:** 10.3390/vaccines13101062

**Published:** 2025-10-17

**Authors:** Chaoying Hu, Haixin Wang, Guanxing Liu, Kelei Li, Xuanxuan Zhang, Lifang Song, Fan Gao, Xing Wu, Qian Wang, Mingchen Liu, Jianyang Liu, Zhihao Fu, Xiao Ma, Miao Xu, Qunying Mao, Zhenglun Liang, Qian He

**Affiliations:** 1 State Key Laboratory of Drug Regulatory Science, Evaluation of Biological Products, Key Laboratory of Research on Quality and Standardization of Biotech Products, Institute of Biological Products, National Institutes for Food and Drug Control, Beijing 102600, Chinaxumiaobj@126.com (M.X.); maoqunying@126.com (Q.M.); 2 Beijing Minhai Biotechnology Co., Ltd., Beijing 102600, China; 3 Changchun Institute of Biological Products Co., Ltd., Changchun 130012, China

**Keywords:** self-amplifying mRNA, design of experiment, integrity, design space, in vitro transcription

## Abstract

Background: Self-amplifying mRNA (saRNA) holds promising application prospects. However, due to the inclusion of a replicase sequence, its extended length leads to premature termination during in vitro transcription (IVT), resulting in poor product integrity. This study aims to optimize the IVT process for saRNA vaccines to enhance integrity, thereby addressing the key challenge in saRNA vaccine manufacturing. Method: Guided by the Quality by Design (QbD) framework, Design of Experiment (DoE) methodology was employed to design diverse combinations of process parameters for IVT reactions. Predictive models were established to identify critical process parameters (CPPs) influencing integrity and yield. An optimized parameter set and process design space, meeting predefined yield and integrity standards, were developed. The impact of integrity on the immunogenicity of saRNA vaccines was further investigated. Results: Mg^2+^ concentration exerted the most pronounced effect on saRNA integrity. Under optimized IVT conditions, integrity exceeded 85%. Mathematical modeling simulations defined the IVT design space, meeting the preset criteria of ≥80% integrity and ≥600 μg/100 μL yield while accommodating longer saRNA constructs. Notably, murine model data revealed that higher saRNA integrity significantly enhanced antigen-specific antibody and T-cell responses. Conclusion: This study successfully established a multivariate IVT design space fulfilling preset integrity and yield criteria, providing critical data references for the industrialization and quality specification development of saRNA vaccines.

## 1. Introduction

Two primary mRNA vaccine platforms exist: conventional non-replicating mRNA and self-amplifying mRNA (saRNA). saRNA vaccines constitute an emerging linear mRNA technology with broad therapeutic potential against infectious and oncological diseases. Currently, research on saRNA vaccines is underway for infectious diseases including COVID-19, influenza, respiratory syncytial virus (RSV), rabies, Ebola, and HIV, as well as for cancer vaccines targeting malignancies such as melanoma [1,2,3], Notably, Japan’s Ministry of Health, Labour, and Welfare (MHLW) recently approved ARCT-154, the first saRNA COVID-19 vaccine for adults [4], and the ARCT-154 vaccine also received approval from EMA on 12 February 2025 [5].

saRNA vaccines integrate viral RNA-dependent RNA polymerase (RdRp) sequences, enabling intracellular self-replication [6]. Following cellular entry, host ribosomes translate saRNA into RdRp complexes. As positive-sense RNAs, saRNA templates negative-strand synthesis by RdRp. These negative strands then direct the production of two positive-sense RNAs: full-length genomic copies and sub-genomic RNAs encoding target antigens [7,8,9,10]. Compared with conventional mRNA vaccines, saRNA offers dose-sparing potential and enhanced immunogenicity. Despite its complex replication mechanism, saRNA shares identical production workflows with non-replicating mRNA: plasmid production, linearization, in vitro transcription (IVT), mRNA purification, and lipid nanoparticle (LNP) encapsulation [4,11]. IVT represents the most critical step, involving multiple parameters that profoundly impact RNA quality, such as template input, nucleotide concentrations, enzyme amount, PH, reaction temperature, and reaction time. This reaction employs T7 promoter-containing DNA templates, where T7 RNA polymerase synthesizes mRNA complementary to the template via ribonucleotide (NTP) incorporation [12]. Crucially, the self-amplifying activity of mRNA is dependent on the encoded RdRp, which results in an inherently longer sequence. Conventional IVT systems optimized for shorter mRNAs often yield inadequate integrity for lengthy saRNA transcripts, severely limiting technological translation. Thus, optimizing IVT processes for long-sequence saRNA to enhance integrity is imperative.

The Quality by Design (QbD) framework, formally introduced by the FDA (2003) and later integrated into ICH guidelines Q8-Q10 [13,14,15,16], represents a proactive development philosophy distinct from reactive approaches like Quality by Test (QbT) or Quality by Production (QbP) [17,18]. QbD mandates quality consideration from development inception. Design of Experiments (DoE), a key QbD implementation tool, systematically evaluates how multiple input variables (factors) collectively affect output variables (responses) through strategically designed trials, replacing inefficient one-factor-at-a-time methods [19]. This enables the identification of how material attributes and process parameters influence critical quality attributes (CQAs), facilitating optimal process conditions and design space establishment [20,21]. In 2021, A study about QbD on mRNA IVT characterized critical process parameter (CPP) effects on CQAs for both short RNA and saRNA, yet failed to establish a high-integrity, high-yield IVT system for long mRNAs [22].

This study aims to develop an saRNA vaccine and optimize its IVT process using QbD principles. Objectives include (1) establishing an IVT process meeting predefined saRNA integrity and yield criteria, and (2) systematically evaluating how varying RNA integrity levels impact vaccine immunogenicity.

## 2. Materials and Methods

### 2.1. Cells and Animals

The human embryonic kidney cell line 293 T used in this study was purchased from ATCC and stocked in our laboratory. The liver cancer cell line HuH-7 used in this study was stocked in our laboratory. In total, 293 T cells and HuH-7 cells were cultured in Dulbecco’s modified eagle medium (DMEM) supplemented with 10% fetal bovine serum (FBS) and 1% penicillin/streptomycin in a humidified incubator at 37 °C with 5% CO_2_. All cell culture reagents were purchased from Gibco. SPF-grade BALB/cmice (6–8-week-old, female, 18–22 g) were provided by and housed at the Laboratory Animal Resource Center, National Institute for Food and Drug Control and were used as experimental animals.

### 2.2. Design and Construction of saRNA Vaccine

The genomic sequence of VEEV-TC83 (GenBank accession no.: MZ399798.1) and the signal peptide sequence and receptor-binding domain (RBD) sequence of the SARS-CoV-2 JN.1 strain S protein (GenBank accession no.: XBC23597.1) were downloaded. Codon optimization was performed for the SARS-CoV-2 JN.1 strain S protein signal peptide sequence and S protein RBD sequence. The pcDNA3.1+ vector was selected to facilitate subsequent synthesis and verification of saRNA. All sequences were assembled and submitted to Sangon Biotech (Shanghai, China) Co., Ltd. for full-length gene synthesis.

### 2.3. Synthesis of saRNA

Plasmids were digested with the restriction enzyme XbaI, and the linearization of plasmid DNA was assessed using 1% agarose gel electrophoresis. The completely linearized DNA template was purified using the Microcolumn DNA Gel Extraction kit (ZomanBiot, Beijing, China; ZPV202). Purified linearized DNA template was used for the IVT of saRNA. After IVT completion, DNase I was added and digested for 30 min. An equal volume of 8 M LiCl was added, mixed thoroughly, and stored at −80 °C for 30 min. The mixture was then centrifuged at 12,000 rpm for 30 min at 4 °C, washed once with 70% anhydrous ethanol, air-dried, and dissolved in an appropriate volume of nuclease-free water.

### 2.4. Detection of Protein Expression by Western Blot

A total of 293 T cells were seeded (1 × 10^6^ cells/well) in 6-well plates until 90% confluency; next, 1 μg, 2 μg, and 5 μg of self-amplifying RNA with an encoding receptor-binding domain (RBD) sequence of the SARS-CoV-2 JN.1 strain (Sa-RBD) were transfected into the 6-well plates with Lipofectamine RNAiMAX reagent (Thermo Fisher scientific, MA, USA; 13778075). At 36 h, cells and cell culture supernatants were collected. Cells were centrifuged, had the supernatant discarded, were washed three times with 1× PBS, and were lysed in 100 μL of lysis buffer on ice for 30 min. The lysate was centrifuged at 12,000 rpm for 30 min, and the supernatant was collected. Protein quantification was performed according to the instructions of the BCA Protein Assay kit (Thermo Fisher Scientific, MA, USA; 23225). Based on the BCA quantification result, an appropriate volume of protein loading buffer was added to the supernatant. The mixture was denatured in boiling water for 10 min. Proteins were separated on a 10% SDS-polyacrylamide gel. An appropriate volume of sample was loaded and electrophoresed at 80 V through the stacking gel and 120 V through the separating gel. Protein transfer was performed using the eBlot™ L1 Rapid Wet Transfer System. Membranes were blocked with 5% skimmed milk at room temperature for 2 h. Primary antibody was added and incubated overnight at 4 °C. Membranes were washed three times with 1× PBST, and a secondary antibody was added and incubated at room temperature for 1 h. Membranes were washed three times with 1× PBST, followed by ECL chemiluminescence detection for signal acquisition.

### 2.5. Detection of the Potein Expression by ELISA

In total, 293 T cells were seeded (1 × 10^6^ cells/well) in 6-well plates until 90% confluency; next, 5 μg of Sa-RBD and linear mRNA were transfected into the 6-well plates with Lipofectamine RNAiMAX reagent (Thermo Fisher scientific, MA, USA; 13778075). At 36 h, cells and cell culture supernatants were collected. Cells were centrifuged, had the supernatant discarded, were washed three times with 1× PBS, and were lysed in 100 μL of lysis buffer on ice for 30 min. The lysate was centrifuged at 12,000 rpm for 30 min, and the supernatant was aspirated. The antibody (Sinobiological; 40592) was diluted to 10 μg/mL in coating buffer, and 100 μL was added per well for overnight coating at 4 °C. Blocking buffer was added and incubated at 37 °C for 2 h. After washing the plate, samples and serially diluted standards were added and incubated at 37 °C for 1 h. After washing, detection antibody was added and incubated at 37 °C for 1.5 h. After washing, horseradish peroxidase (HRP)-conjugated secondary antibody (Zhongshan Golden Bridge Biotechnology; ZB-2305) was added and incubated at 37 °C for 1 h. After washing, freshly prepared chromogenic substrate (Wantai BioPharm; ZDT0175, ZDT0181) was added, developed in the dark for 15 min, and then stop solution (Wantai BioPharm; ZDT0178) was added. The plate was immediately read at dual wavelengths of 450/630 nm.

### 2.6. Detection of Target RNA Relative Content Using qPCR

A total of 293 T cells were seeded (1 × 10^6^ cells/well) in 6-well plates until 90% confluency; next, 5 μg of Sa-RBD and linear mRNA were transfected into the 6-well plates. The culture medium was replaced at 12 h post-transfection, and cells were collected at 12 h, 24 h, 36 h, 48 h, 60 h, 72 h, 84 h, 96 h, 108 h, and 132 h post-transfection, respectively, washed three times with PBS, and lysed in 100 μL of lysis buffer. qPCR was performed using the One Step TB Green^®^ PrimeScript™ RT-PCR kit II (Takara, Beijing, China; RR086A), according to the manufacturer’s instructions. The target RNA content at 12 h was set as 100%, and results were relatively quantified using the 2^(−ΔΔCt)^ method.

### 2.7. DoE Experimental Design and Data Analysis

The Design of Experiments (DoE) for optimizing self-amplifying mRNA IVT process parameters was designed using JMP software (JMP Pro 17). A Central Composite Design (CCD) within Response Surface Methodology (RSM) was employed to generate the experimental design. Experimental data were obtained according to the design and analyzed using JMP software (JMP Pro 17).

### 2.8. Detection of saRNA Integrity and Yield

Self-amplifying mRNA obtained from the IVT experiments according to the DoE design was assessed for integrity and yield. The concentration of self-amplifying mRNA obtained from IVT was measured using NanoDrop. The integrity of self-amplifying mRNA was detected using the Qsep100 Fully Automated Capillary Electrophoresis System (BIOptic) with an N3 cartridge, with the 9000 nt RNA Ladder analyzed simultaneously with the samples.

### 2.9. Preparation of saRNA Vaccines

Sa-RBD with 37%, 64%, and 80% integrity, obtained from process optimization, was encapsulated using microfluidic mixing technology (MIANANO, INano L, Shanghai, China) with a lipid mixture (SM102). The encapsulated product was buffer-exchanged by ultrafiltration, and an equal volume of 20% sucrose buffer was added. Self-amplifying mRNA vaccines with 37%, 64%, and 80% integrity were named 0.37 V-Sa-RBD, 0.64 V-Sa-RBD, and 0.80 V-Sa-RBD, and stored at −20 °C. The encapsulation efficiency of the vaccines was detected using the Quant-iT™ RiboGreen™ RNA reagent and kit (Invitrogen, MA, USA; R11490). The average particle size and polydispersity index (PDI) of the vaccines were detected using the Malvern Zetasizer Nano ZS (Malvern, UK).

### 2.10. Immunization Sample Collection and Processing

Blood was collected from the retro-orbital venous plexus of mice at 2 weeks after the first immunization and 5 weeks after the second immunization, with 200 μL collected per mouse. The collected whole blood was left at room temperature for 2 h and centrifuged at 6000 rpm for 10 min; the serum was collected and stored at −20 °C. Spleens were ground in RPMI-1640 complete medium, and the spleen homogenate was collected. The homogenate was centrifuged at 500× *g* for 10 min, the supernatant was discarded, and the pellet was gently resuspended. Three milliliters of red blood cell lysis buffer were added, mixed by inversion, and lysed for 3 min. An equal volume of RPMI-1640 complete medium was added to stop the lysis, the solution was centrifuged at 500× *g* for 10 min, and the supernatant was discarded. Splenocytes were resuspended in 3 mL of cell freezing medium, frozen using a gradient freezing protocol, and ultimately stored at −80 °C.

### 2.11. ELISA for Spike-Specific IgG

Spike-specific IgG antibodies were detected using ELISA. The spike proteins of JN.1 were individually coated at 1 µg/mL on 96-well plates overnight at 4 °C. The subsequent methods were performed as described in Section 2.5.

### 2.12. Pseudovirus Neutralization Assay

To avoid edge effects, 250 μL of cell maintenance medium was added to the peripheral wells of a 96-well culture plate. Row 2 received 142.5 μL of cell maintenance medium. Cell control wells received 150 μL of cell maintenance medium, virus control wells received 100 μL of cell maintenance medium, and the remaining wells received 100 μL of cell maintenance medium. Test serum was pre-diluted in the cell maintenance medium at specific dilutions, and 7.5 μL per well (in duplicate) was added to row 2 of the 96-well culture plate, followed by three-fold serial dilution. Fifty microliters of 1 × 10^4^ TCID_50_/mL SARS-CoV-2 pseudovirus were added to each well, mixed thoroughly, and incubated at 37 °C, 5% CO_2_ for 1 h for neutralization. HuH-7 cell suspension was diluted to 2 × 10^5^ cells/mL, and 100 μL was added per well. The plate was incubated at 37 °C, 5% CO_2_ for 20–24 h. Subsequently, 150 μL of cell maintenance medium was aspirated, 100 μL of luciferase detection reagent was added, and luminescence was measured using a high-throughput multi-mode microplate detection system.

### 2.13. Enzyme-Linked Immunospot (ELISpot) Assay for IFN-γ

ELISpot plates were coated with purified anti-mouse IFN-γ antibody (BD Biosciences; 51-2525KC) and incubated overnight at 4 °C. Plates were washed with DMEM complete medium and blocked with DMEM complete medium at room temperature for 2 h. After discarding the blocking solution, 50 μL of 10 μg/mL peptide pool and 50 μL of cells (4 × 10^6^/mL) were added, and incubated at 37 °C, 5% CO_2_ for 20 h. Plates were washed twice with pre-chilled deionized water and three times with 1× PBST. Biotinylated Detection antibody (BD Biosciences; 51-1818KZ) was diluted 1:250 in antibody dilution buffer, and 100 μL was added per well to the ELISpot plate, incubated at room temperature for 2 h. Plates were washed three times with 1× PBST. Streptavidin–HRP was diluted 1:100 in antibody dilution buffer, and 100 μL was added per well to the ELISpot plate, incubated at room temperature for 2 h. Plates were washed four times with 1× PBST and twice with 1× PBS, blotted dry, and 100 μL of chromogenic substrate was added per well for 10 min. The reaction was stopped by washing with deionized water, air-dried at room temperature, and spots were counted using an automated ELISpot reader (CTL-ImmunoSpot^®^ S6, Cleveland, OH, USA).

### 2.14. Data Processing

Statistical analysis of all experimental results was performed using GraphPad Prism 9 (GraphPad Software Inc., USA). T-test was used to compare statistical differences between two groups. Analysis of variance (ANOVA) was used to compare statistical differences among multiple groups. *p* < 0.05 was considered statistically significant and denoted by _*_, *p* < 0.01 was considered significantly different and denoted by **, *p* < 0.001 was considered highly significantly different and denoted by ***, and *p* < 0.0001 was considered extremely highly significantly different and denoted by ****. JMP software was used for IVT experimental design and data model fitting.

## 3. Results

### 3.1. Construction of saRNA Encoding RBD

**Figure 1:**
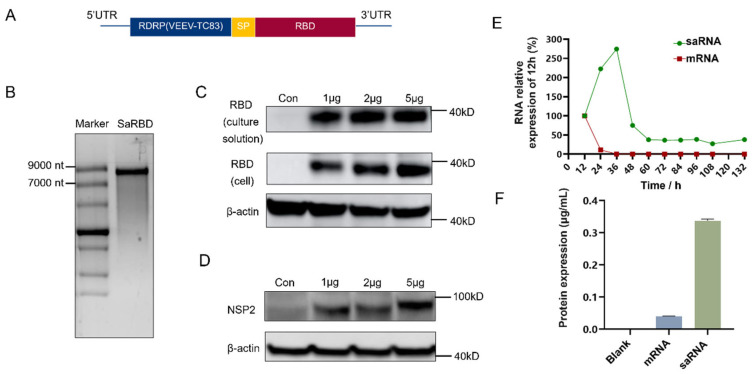
Construction of self-amplifying mRNA expressing RBD. (**A**) Sequence design of saRNA Sa-RBD. (**B**) Agarose gel electrophoresis of IVT product Sa-RBD. (**C**) Western blot analysis for RBD protein expression after Sa-RBD transfection. (**D**) Western analysis for NSP2 protein expression after Sa-RBD transfection. (**E**) Relative expression level of target RNA in self-amplifying mRNA Sa-RBD and non-replicating mRNA at different time points compared with the 12 h level. (**F**) Target protein expression in 293 T cells transfected with Sa-RBD and non-replicating mRNA was detected by ELISA (3 replicates; error bars indicate the standard deviation).

The saRNA sequence (Figure 1A) was ligated into the pcDNA3.1(+) vector to construct the plasmid (P-Sa-RBD), and the linearized template (L-P-Sa-RBD) was obtained. The proportion of supercoiled plasmid and the purity of the linearized template were detected (Figure S1A–C). Using this system, we synthesized saRNA expressing EGFP with different base modifications [23,24,25,26]. Unmodified saRNA demonstrated stronger and longer-lasting expression in 293 T cells (Figure S2). Therefore, we used the unmodified IVT system to obtain saRNA encoding RBD (Sa-RBD) (Figure 1B). The expression of RBD protein and NSP2 protein was detected by Western blot 36 h post-transfection with Sa-RBD (Figure 1C,D). At the same transfection dose of 5 μg, the RBD RNA content in saRNA-transfected cells showed a self-amplifying trend—increasing initially, then decreasing and stabilizing—whereas cells transfected with non-replicating mRNA exhibited no such change in RBD RNA content (Figure 1E). At 36 h post-transfection with equal doses, the RBD protein level in saRNA-transfected cells was significantly higher than in non-replicating mRNA-transfected cells (Figure 1F). These results indicate that the saRNA backbone confers both expression and self-amplification capabilities.

### 3.2. DoE-Designed IVT Experiments

Response Surface Methodology (RSM) was selected for experimental design to optimize the IVT process for saRNA vaccines. The integrity and yield of the IVT product were set as the response variables for each experiment. Based on prior knowledge, five parameters potentially affecting IVT integrity and yield were initially identified. The levels for each factor were set as follows: Mg^2+^ concentration (6~75 mM), T7 RNA polymerase concentration (5~10 U/μL), IVT reaction time (2~4 h), cap analog concentration (4~10 mM), and DNA template input amount (30~60 ng/μL). Other IVT parameters were fixed at NTPs (10 mM), 1 M Tris-HCl (40 mM), 1 M DTT (10 mM), spermidine (2 mM), pyrophosphatase (0.002 U/μL), and RNase inhibitor (1 U/μL). Central Composite Design (CCD) can more accurately describe the relationship between response variables and factors. Therefore, a multi-factor CCD was implemented using JMP software across defined levels for each factor, yielding a total of 28 IVT experiments (Table S1).

**Figure 2:**
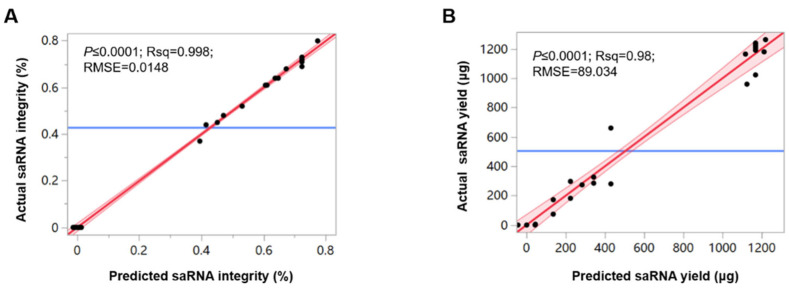
Prediction** **models for self-amplifying mRNA SaRBD integrity and yield. (**A**) Predicted vs. actual values based on the integrity model. (**B**) Predicted vs. actual values based on the yield model.

The integrity and yield of the obtained saRNA were detected. Prediction models for integrity and yield were fitted using the Least Squares Method. For the saRNA integrity model: Root Mean Square Error (RMSE) = 0.0148, R-squared (R^2^) = 0.99838, *p* = <0.0001 (Figure 2A). For the self-amplifying mRNA yield model: RMSE = 89.034, R^2^ = 0.98, *p* = <0.0001 (Figure 2B).

### 3.3. Main Effects and Interaction Effects of Different IVT Reaction Parameters on Integrity and Yield

**Figure 3:**
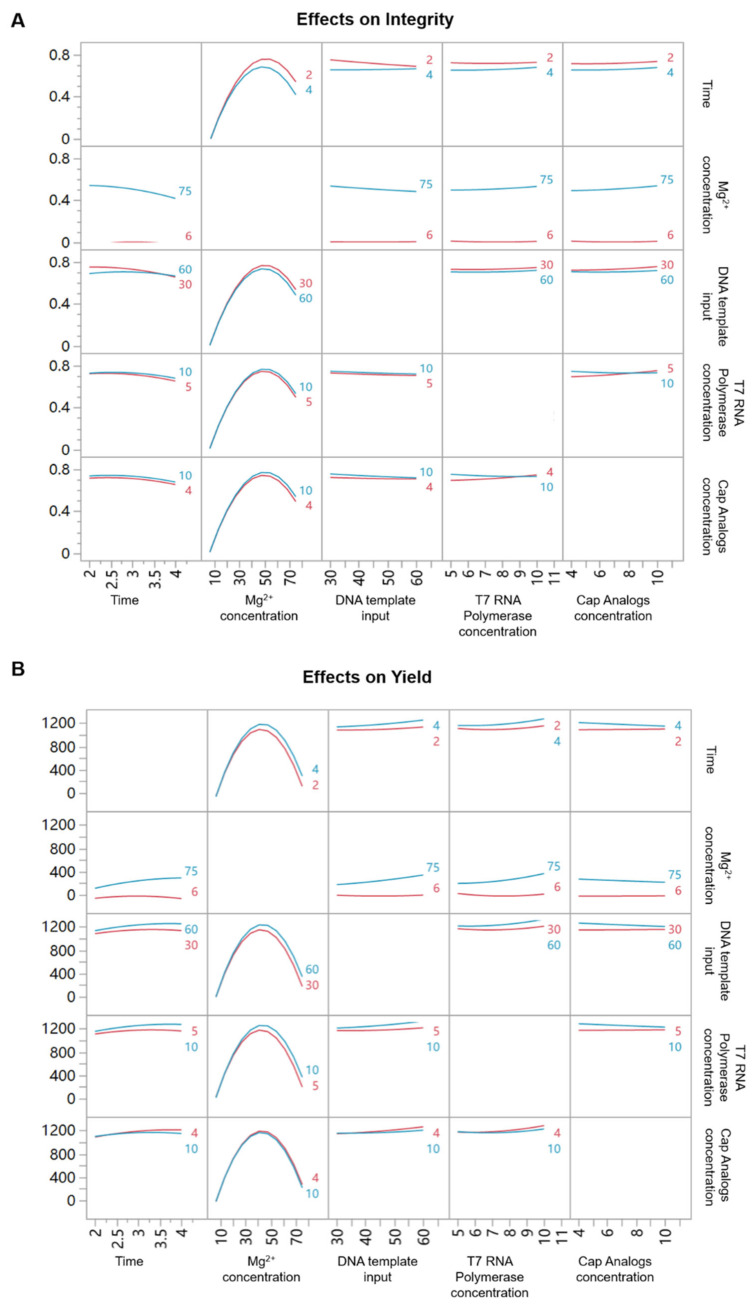
Parameter effects on integrity and yield of saRNA IVT products. (**A**) Interaction plot of five variables on the integrity of saRNA IVT products. (**B**) Interaction plot of five variables on the yield of saRNA IVT products. The red lines represent the upper boundary of parameter range, while the blue lines represent the lower boundary.

JMP software was used to analyze the effects and interactions of different factors on integrity and yield, obtaining LogWorth values for each factor to reflect their contributions to the predictive model (Table 1). In the predictive model for saRNA integrity, Mg^2+^ concentration (*p* < 0.00001, LogWorth = 10.017) and IVT reaction time (*p* < 0.00001, LogWorth = 3.685) had the most significant effects on saRNA integrity, and these two exerted interaction effects (*p* < 0.00001, LogWorth = 3.740). The effect of Mg^2+^ concentration on saRNA integrity was non-linear. Under different IVT reaction time conditions, as Mg^2+^ concentration gradually increased within the 6~75 mM range, the integrity of IVT products all showed an initial increase followed by a decrease. When Mg^2+^ concentration was 6 mM, IVT reaction time had no significant effect on integrity, and integrity was almost 0; but when Mg^2+^ concentration rose to 75 mM, integrity significantly decreased with increasing IVT time. According to the above data, it can be concluded that under lower IVT reaction time conditions, selecting moderate Mg^2+^ concentration and lower T7 polymerase can obtain IVT products with higher integrity, conserving more materials and time (Figure 3A).

In the predictive model for self-amplifying mRNA yield, Mg^2+^ reaction concentration (*p* = 0.0001, LogWorth = 3.987), IVT reaction time (*p* = 0.03713 LogWorth = 1.430) and DNA template input amount (*p* = 0.4082, LogWorth = 1.389) had significant effects on saRNA yield. The effects of the DNA template input amount and Mg^2+^ reaction concentration on self-amplifying mRNA yield had interaction effects (*p* = 0.05427, LogWorth = 1.265). Similarly to the integrity model, the effect of Mg^2+^ reaction concentration on self-amplifying mRNA yield was non-linear. Under different DNA template input amount conditions, as Mg^2+^ reaction concentration gradually increased within the 6 mM~75 mM range, the integrity of IVT products all showed an initial increase followed by a decrease. When Mg^2+^ concentration was at 6 mM, the yield was not affected by the DNA template input amount; but when Mg^2+^ reaction concentration rose to 75 mM, yield increased with increasing DNA template input amount. The above data indicate that selecting moderate Mg^2+^ reaction concentration and higher DNA template input amount helps improve the yield of saRNA (Figure 3B).

### 3.4. The Optimal IVT System and Multivariate Design Space

Using JMP software, the two response variables (yield and integrity) were fitted for grouped data analysis. Through desirability function settings, the importance of integrity was set to 3 and yield to 1. The optimal process parameters were obtained: a DNA template input amount of 60 ng/μL, Mg^2+^ concentration of 52 mM, IVT reaction time of 2 h, cap analog concentration of 10 mM, and T7 RNA polymerase concentration of 5 U/μL. Under these parameters, IVT was expected to obtain a yield of 1121.5 μg and integrity of 86.5% (Table 2).

The design space is a multivariate combination and interaction range of input variables and process parameters that has been proven to ensure product quality. Operating within this space ensures the quality meets preset criteria. Based on product quality requirements and process capability, the criterion for self-amplifying mRNA integrity was set at 80%, and the criterion for yield was set at 600 μg/100 μL. Monte Carlo Simulation was performed to obtain the process parameter design space meeting this quality criteria. Results showed the design space: DNA template input amount of 50~60 ng/μL, Mg^2+^ concentration of 50~55 mM, IVT reaction time of 2~2.2 h, cap analog concentration of 7~10 mM, T7 RNA polymerase concentration of 5~7.5 U/μL (Table 3). The defect rate was 0% after 10,000 simulations of this model.

**Figure 4:**
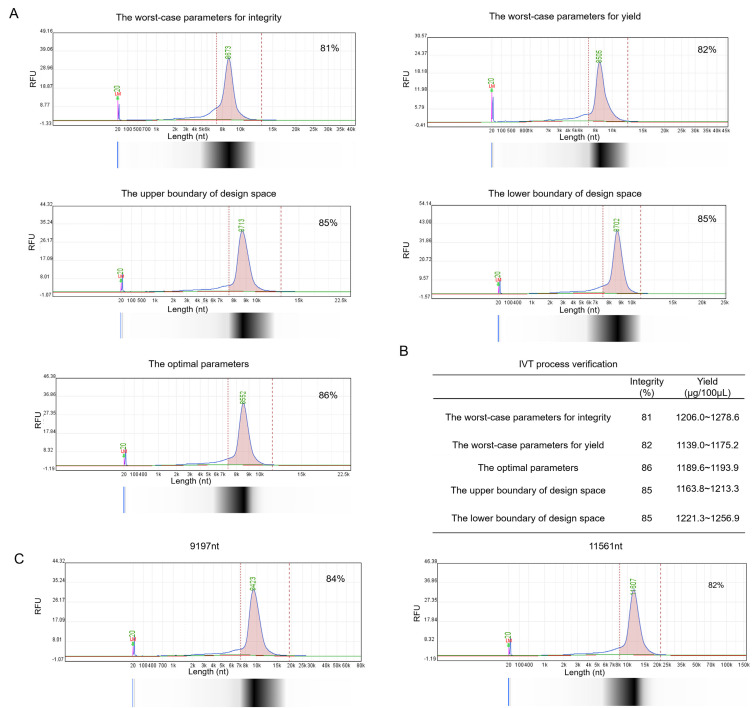
Verification** **of optimal IVT parameters and design space effects on saRNA integrity. (**A**) Capillary gel electrophoresis (CGE) analysis of saRNA integrity under five IVT conditions: worst-case parameters for integrity, worst-case parameters for yield, upper design space boundary, lower design space boundary, optimal IVT parameters. (**B**) Summary of verification results. (**C**) Integrity analysis of extended length saRNA IVT products (Length variants: 9197 nt vs. 11,561 nt).

Generally, if the verification results of the extreme worst-case combination parameters from the design space meet the preset criteria, the design space is considered compliant with production requirements. Verification was conducted according to the extreme worst-case combination parameters, the optimal process parameters, the upper and lower boundary combinations of the design space. All results satisfied the preset criteria (Figure 4A,B). Additionally, we applied the optimal process parameters to prepare longer saRNA sequences (9.2k nt, 11.5k nt) to confirm its applicability for longer saRNA. The results showed that IVT products could still meet the preset criteria (Figure 4C).

### 3.5. Immunogenicity of Sa-RBD Vaccines with Different Integrities in Murine Model

**Figure 5:**
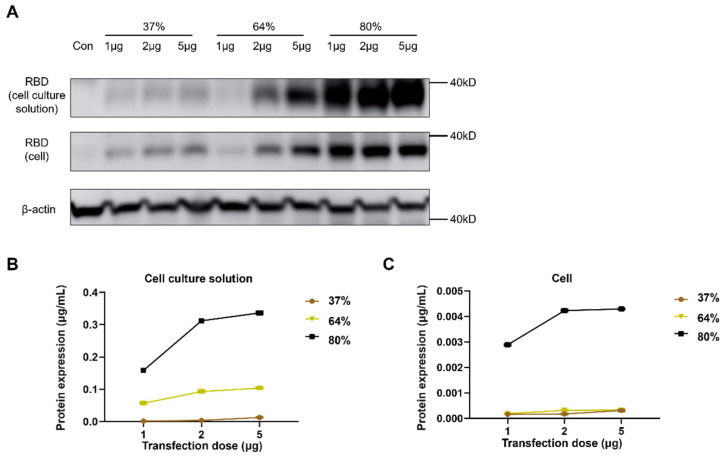
In vitro expression efficiency of Sa-RBD correlates with saRNA integrity. (**A**) Western blot analysis of target protein expression in 293 T cell lysates and cell culture supernatants after transfection with Sa-RBD of different integrities. (**B**) ELISA quantification of target protein in culture supernatants from transfected 293 T cells. (**C**) Intracellular target protein expression in transfected 293 T cells detected by ELISA.

Cells and supernatants were collected 36 h after transfecting 293 T cells with 1 μg, 2 μg, or 5 μg of Sa-RBD at 37%, 64%, or 80% integrity. Target protein expression was detected by Western blot. Under different transfection doses, as integrity increased, the target protein bands in both cells and culture supernatants gradually intensified. At all doses, Sa-RBD with 80% integrity exhibited the strongest protein expression (Figure 5A). In addition, dose-escalation cannot sufficiently compensate for reduced expression levels caused by low-integrity Sa-RBD. The amount of target protein in cells and supernatants detected by ELISA showed consistent trends with the Western blot results (Figure 5B,C).

Sa-RBDs with different integrity were encapsulated by LNP to achieves saRNA vaccines. Mice were immunized with three different doses (0.3 μg, 1 μg, 3 μg) of the 80% integrity saRNA vaccine (0.80 V-Sa-RBD). Results showed that, 2 weeks post-immunization, all three dose groups achieved seroconversion for RBD-specific binding antibodies and neutralizing antibodies. Geometric Mean Titers (GMTs) showed a dose–response relationship. The 3 μg single-dose immunization induced the highest serum binding antibodies and neutralizing antibodies at 2 weeks, with binding antibody GMT at 1:55127 and pseudovirus neutralizing antibody GMT at 1:467 (Figure S3). Thus, the 3 μg dose was used for evaluation of the immunogenicity of SaRBD vaccines with different integrities.

**Figure 6:**
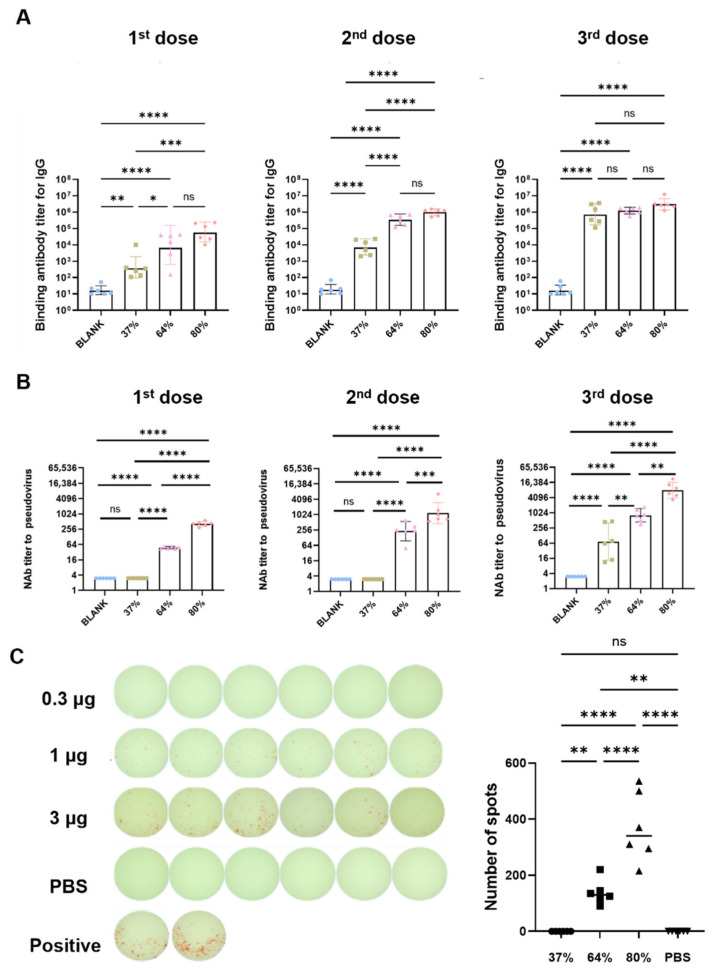
Immunogenicity** **of Sa-RBD vaccines with different integrities. (**A**) Binding antibody titers induced by Sa-RBD vaccines of different integrities 2 weeks after the first immunization, 5 weeks after the second immunization, and 2 weeks after the third immunization (error bars indicate geometric mean with 95% CI). (**B**) Pseudovirus neutralizing antibody titers induced by Sa-RBD vaccines of different integrities 2 weeks after the first immunization, 5 weeks after the second immunization, and 2 weeks after the third immunization (error bars indicate geometric mean with 95% CI). (**C**) IFN-γ-secreting RBD-specific T cells quantified by ELISPOT assay. (*n* = 6, * indicates *p* < 0.05, ** indicates *p* < 0.01, *** indicates *p* < 0.001, **** indicates *p* < 0.0001, ns indicates not significant).

Mice were immunized with 3 μg of saRNA vaccines at 37%, 64%, and 80% integrities (Table S2). Two weeks post-immunization, the seroconversion rate for spike protein-specific binding antibodies was 100% in all groups. The 37% integrity vaccine failed to effectively induce pseudovirus neutralizing antibodies. Pseudovirus neutralizing antibody GMTs for the 64% and 80% integrity vaccines were 1:48 and 1:411, respectively. The pseudovirus neutralizing antibody titer induced by the 80% integrity vaccine was 137-fold higher than that of the 37% integrity vaccine (*p* < 0.0001) and 16.1-fold higher than that of the 64% integrity vaccine (*p* < 0.0001) (Figure 6A,B). The second and third vaccine doses were administered at 2 weeks and 7 weeks after the first immunization, respectively. Five weeks after the second immunization, GMTs for spike protein-specific binding antibodies in the 37%, 64%, and 80% integrity groups were 1:6906, 1:335865, and 1:973253, respectively. Pseudovirus neutralizing antibody GMTs were 1:3, 1:230, and 1:1172, respectively. The pseudovirus neutralizing antibody titer induced by the 80% integrity vaccine was 390.7-fold higher than that of the 37% integrity vaccine (*p* < 0.0001) and the 64% integrity vaccine was 76.7-fold higher than that of the 37% integrity vaccine (*p* < 0.0001). Two weeks after the third immunization, GMTs for spike protein-specific binding antibodies in the 37%, 64%, and 80% groups were 1:696492, 1:1204447, and 1:2982650, respectively. Pseudovirus neutralizing antibody GMTs were 1:73, 1:811, and 1:8083, respectively. The pseudovirus neutralizing antibody titer induced by the 80% integrity vaccine was 110.7-fold higher than that of the 37% integrity vaccine and 9.96-fold higher than that of the 64% integrity vaccine (*p* < 0.01) (Figure 6A,B).

Spleens were harvested 2 weeks after the third immunization to prepare splenocyte suspensions. Splenocytes were stimulated with RBD peptide pools, and the frequency of IFN-γ-secreting T cells was detected. Results showed that the 37% integrity group failed to induce a specific T cell response. The mean spot numbers per 1 × 10^6^ cells for the 37%, 64% and 80% integrity groups were 0, 138, and 370, respectively. The number of IFN-γ spots produced by the 80% integrity group was significantly higher than that of the 64%, 37%, and PBS groups (*p* < 0.0001). These results indicate that integrity severely impacts both the antibody response and the Th1-type T cell response levels induced by saRNA vaccines (Figure 6C).

## 4. Discussion

saRNA vaccines represent an emerging technology that retains the advantages of conventional non-replicating mRNA vaccines while enabling dose reduction and enhanced immunogenicity through intracellular self-amplification [27]. For our saRNA designs, we specifically evaluated the impact of nucleotide modifications on in vitro expression in 293 T cells. As exogenous molecules, unmodified mRNA vaccines activate innate immunity upon delivery, causing inflammation and premature clearance [28]. Pseudouridine (Ψ) or N1-methylpseudouridine (m1Ψ) incorporation attenuates immune stimulation, enhances mRNA stability, and improves protein expression [29,30]. Notably, our findings reveal a critical distinction for saRNA systems: unmodified and 5-methylcytidine -modified saRNA demonstrated sustained, potent protein expression in 293 T cells although the latter showed a lower level, whereas m1Ψ- or Ψ-modified saRNA failed to express target proteins. We propose that m1Ψ/Ψ modifications disrupt replicase–template binding, impairing self-amplification. This aligns with reports that unmodified saRNA achieves high expression in IFN-deficient 293 T cells but not in IFN-competent cells (e.g., HeLa, MRC5) [31] or primary human dendritic cells [32]. We therefore recommend that saRNA vaccine design incorporate comprehensive modification strategy assessment—including type and omission—tailored to vaccination sites and cell types, with emphasis on immunogenicity.

The long length of saRNA often causes IVT premature termination, compromising integrity. Current optimization typically adapts protocols for shorter mRNAs (<2k nt) to longer saRNA constructs, yielding suboptimal results. Our study revealed that such protocols produce saRNA with <60% integrity. Critically, LNP-encapsulated saRNA with varying integrities induced divergent antibody/T-cell responses in mice, confirming its essential role in efficacy. Regulatory frameworks—including the WHO’s Evaluation of the quality, safety and efficacy of messenger RNA vaccines for the prevention of infectious diseases: regulatory considerations [33] and USP’s Analytical Procedures for mRNA Vaccine Quality—Draft guidelines: 2nd Edition [34] —explicitly recognize mRNA structural integrity as a CQA for intermediate and final product release, with specified analytical methodologies.

This study employed a DoE approach to analyze five IVT factors identified via brainstorming: Mg^2+^ concentration, IVT reaction time, DNA template input, T7 RNA polymerase concentration, and cap analog concentration. These factors were considered to potentially have greater impact on IVT yield and integrity. Among all five factors examined, Mg^2+^ concentration most significantly impacted yield and integrity (LogWorth = 10.017), exhibiting non-linear effects: both metrics increased then decreased across 6–75 mM. Mechanistically, Mg^2+^ stabilizes RNA structures and acts as a T7 RNA polymerase cofactor [35,36]. It complexes with NTPs during transcription; subsequent pyrophosphate (PPi) release forms insoluble Mg_2_PPi precipitate, inhibiting transcription. Concurrent H^+^ release lowers pH, suppressing enzyme activity. This explains the observed non-linear relationship. Therefore, we conclude that utilizing a DoE approach to identify the optimal inflection point for Mg^2+^ concentration in the IVT system is crucial for integrity improvement.

Using an 8.5 kb saRNA model, Monte Carlo simulations based on DoE data identified optimal IVT parameters and established a multivariate design space ensuring saRNA >80% integrity. Verifications were conducted using the optimal/worst-case IVT parameters, and the upper/lower boundary of the design space. Results confirmed that both purity and yield met predefined criteria across all verification scenarios. To test generalizability, we synthesized saRNA molecules of longer lengths, including 9.2 kb and 11.5 kb. The optimized IVT system consistently produced high-integrity, high-yield products across all lengths, demonstrating robustness for long-chain saRNA. Integrity is a critical challenge in the IVT synthesis of saRNA. In contrast to previous DOE optimization studies for IVT that primarily focused on yield, this study addresses both yield and integrity, thereby providing more data to support process development.

## 5. Conclusions

This study applied the QbD concept of pharmaceutical development to investigate the IVT process for long-sequence saRNA. Using DoE, we (i) analyzed parameter effects on mRNA integrity/yield, (ii) identified optimal conditions, (iii) established a design space, and iv) demonstrated its applicability across saRNA lengths. By integrating quality considerations at the design stage, this work embodies the concept of proactive process control. Moreover, we investigated the impact of RNA integrity on vaccine immunogenicity, thereby informing the development of quality standards. This study still has certain limitations: only five process parameters identified through brainstorming were investigated, without analyzing the effects of other parameters and their interactions. Furthermore, although residual dsRNA and purity of saRNA products with varying integrity levels were tested and met expected standards in this study, in actual process development, all CQAs of IVT products should be integrated in process design.

## Figures and Tables

**Table 1:** Effects of various reaction components and interactions on the integrity and yield of self-amplifying mRNA IVT.

Parameters and Interactions	LogWorth (Integrity)	LogWorth (Yield)
Mg^2+^	10.017	3.987
Mg^2+^ × Mg^2+^	8.816	7.180
Time × Mg^2+^	3.740	1.428
Time	3.685	1.430
Cap Analog × T7 RNA polymerase	2.328	0.418
DNA template × Time	2.328	0.418
DNA template	1.730	1.389
DNA template × Mg^2+^	1.671	1.265
Cap Analog × Mg^2+^	1.496	0.367
Cap Analog	1.483	0.313
Time × Time	1.308	0.392
T7 RNA polymerase × Mg^2+^	1.137	1.402
T7 RNA polymerase	0.975	1.299
T7 RNA polymerase × Time	0.609	0.431
DNA template × Cap Analog	0.609	0.431
T7 RNA polymerase × T7 RNA polymerase	0.298	0.383
Cap Analog × Cap Analog	0.298	0.010
DNA template × DNA template	0.098	0.136
DNA template × T7 RNA polymerase	0.050	0.468
Cap Analog × Time	0.050	0.468

**Table 2:** Optimal IVT parameters and expected integrity and yield.

IVT System		Value
Parameters	DNA Template (ng/μL)	60
	Mg^2+^ (mM)	52
	Time (h)	2
	Cap Analog (mM)	10
	T7 RNA Polymerase(U/μL)	5
Yield	Mean (95% CI) (μg)	1121.5 (1038.2, 1204.8)
Integrity	Mean (95% CI) (%)	86.5 (84.4, 88.6)

**Table 3:** The design space for IVT reaction.

Parameters	Upper Boundary	Lower Boundary
DNA Template (ng/μL)	60	50
Mg^2+^ (mM)	55	50
Time (h)	2.2	2
Cap Analog (mM)	10	7
T7 RNA Polymerase(U/μL)	7.5	5

## Data Availability

The data that support the findings of this study are available on request from the corresponding author (Q.H.) upon reasonable request.

## Supplementary Materials

The following supporting information can be downloaded at https://www.mdpi.com/article/10.3390/vaccines13101062/s1, Table S1: CCD Experimental Plan and Response Value; Table S2. Encapsulation efficiency and Z-average Size and PDI of Sa-RBD vaccines with different integrities; Figure S1: Characterization of plasmid and linearized DNA templates; Figure S2: GFP expression in 293 T cells transfected with Sa-EGFP mRNA containing distinct nucleotide modifications; Figure S3: Dose-dependent immune responses to Sa-RBD vaccine (80% integrity).
