# Supplementary material for: Optimization of In Vitro Transcription by Design of Experiment to Achieve High Self-Amplifying RNA Integrity
Source: Vaccines (Basel). 2025 Oct 17;13(10):1062. doi: 10.3390/vaccines13101062 (PMC12567852; doi:10.3390/vaccines13101062)
Supplement: Supplementary file 1 [file vaccines-13-01062-s001.zip › vaccines-3902089-supplementary.pdf]

Supplementary materials

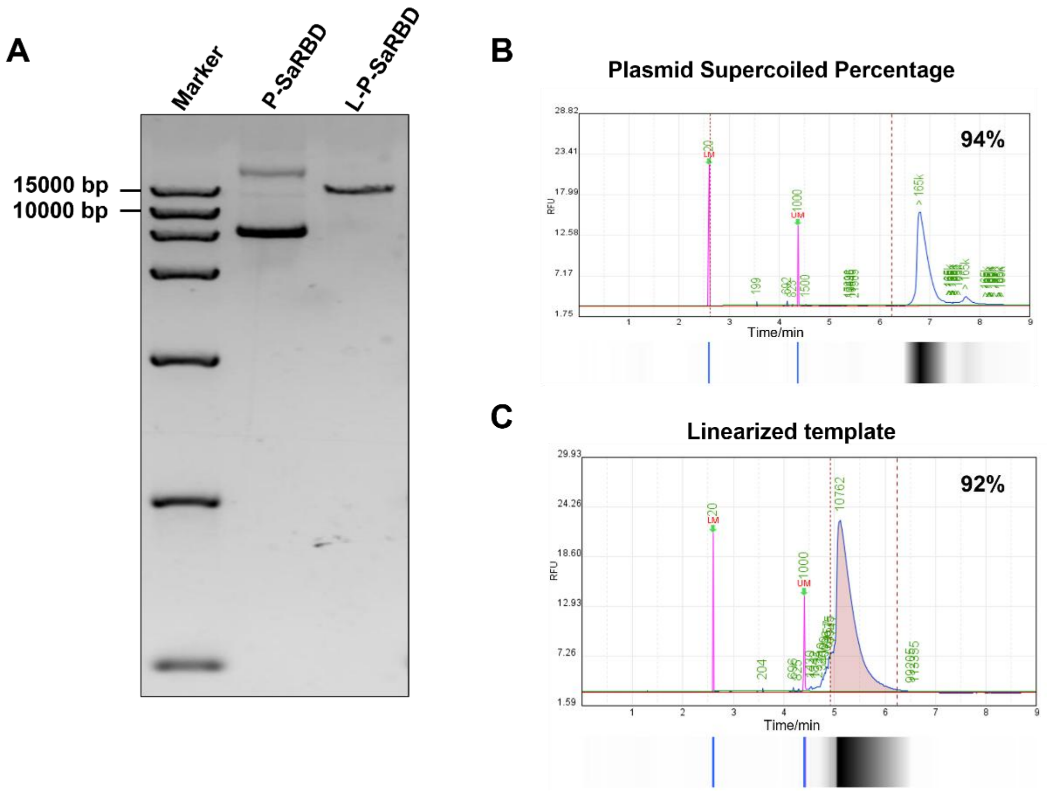

Figure S1. Characterization of plasmid and linearized DNA templates. (A) Agarose gel electrophoresis analysis of plasmid and linearized template DNA. (B) CE assessment of supercoiled plasmid content. (C) CE analysis of linearized template purity.

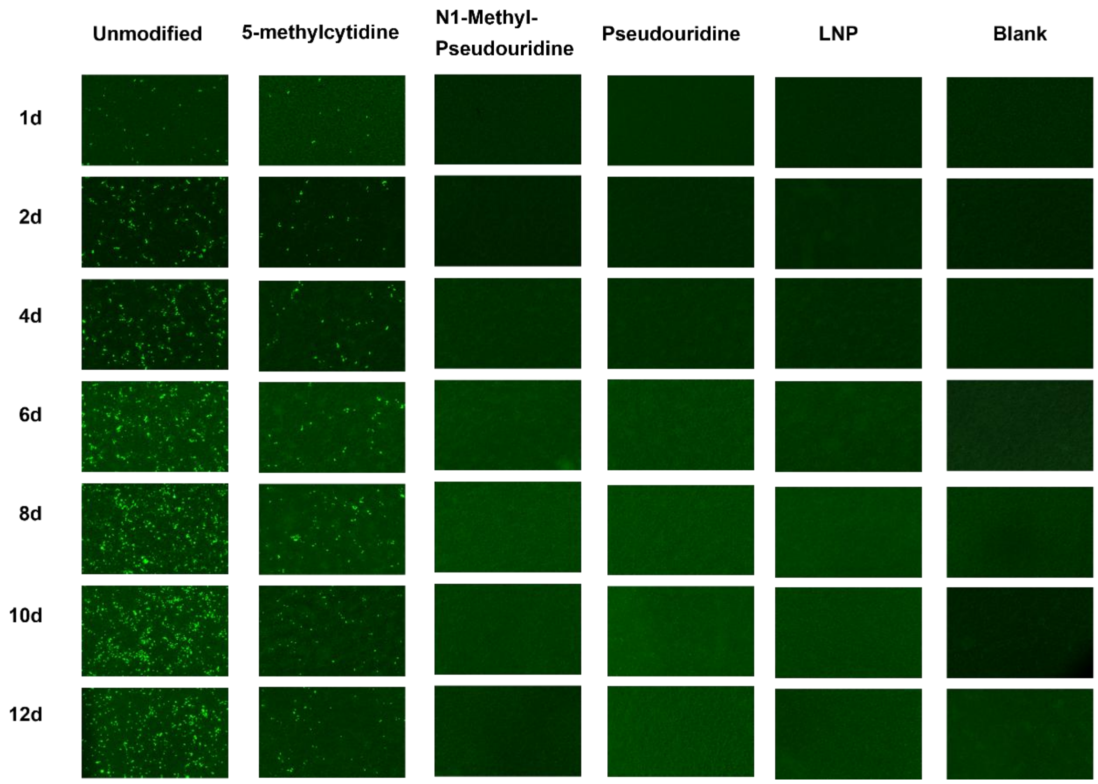

Figure S2. GFP expression in 293T cells transfected with Sa-EGFP mRNA containing distinct nucleotide modifications (Note: The RNA dose used was 5 μg, and the transfection was performed following the protocol described in Section 2.5.)

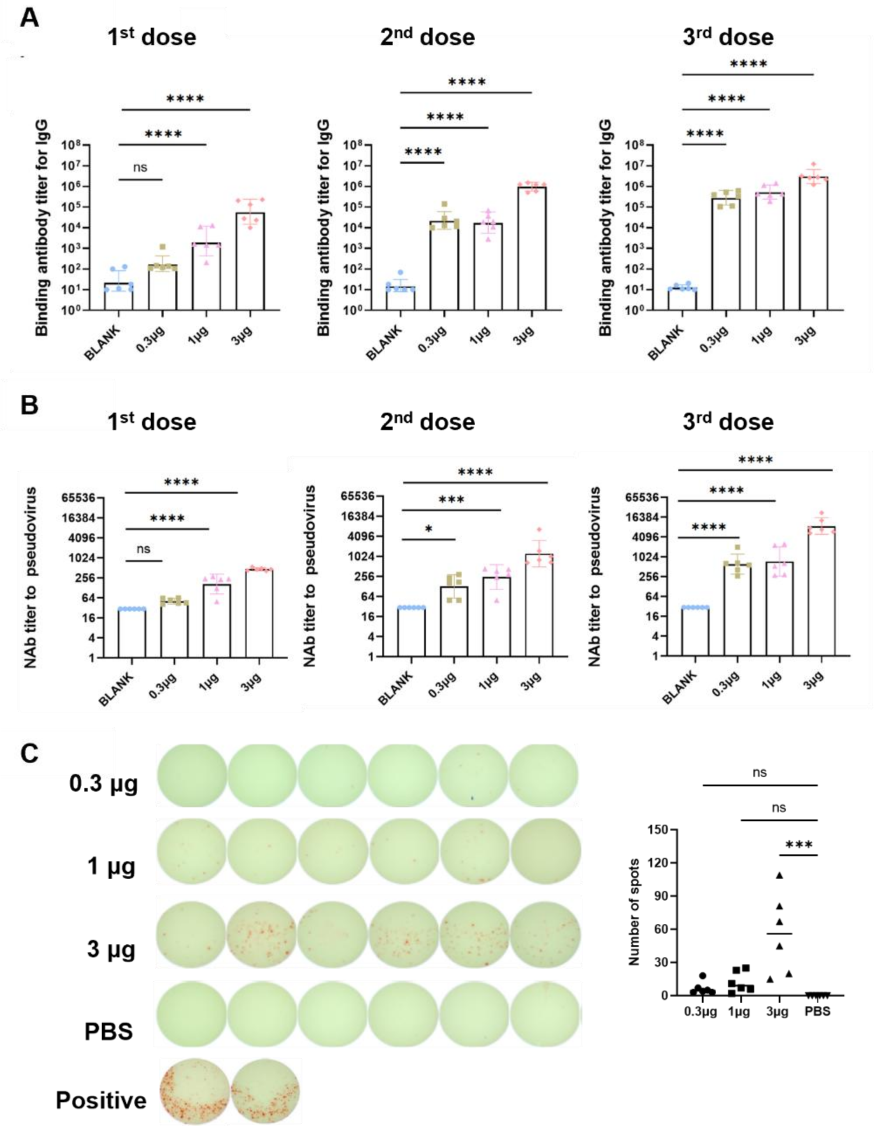

Figure S3. Dose-dependent immune responses to Sa−RBD vaccine (80% integrity). (**A**) Binding antibody results induced 2 weeks after the first immunization, 5 weeks after the second immunization, and 2 weeks after the third immunization. (Error bars indicate geometric mean with 95% CI) (**B**) Pseudovirus neutralizing antibody results induced 2 weeks after the first immunization, 5 weeks after the second immunization, and 2 weeks after the third immunization. (Error bars indicate geometric mean with 95% CI) (C) IFN-γ-secreting RBD-specific T cells quantified by ELISPOT assay. (n=6, * indicates $p < 0.05$, ** indicates $p < 0.01$, *** indicates $p < 0.001$, **** indicates $p < 0.0001$).

Table S1 CCD Experimental Plan and Response Value

No.	DNA Template （ng/μL）	Cap Analog （mM）	T7 RNA Polymerase （U/μL）	Mg^{2+} （mM）	Reaction Time （h）	Yield （μg）	Integrity （%）
1	30	4	10	6	4	0.17	0
2	45	7	7.5	6	3	0.06	0
3	45	10	7.5	40.5	2	958.9	80
4	60	10	10	6	4	0.88	0
5	60	4	10	75	4	660.35	45
6	45	7	7.5	40.5	4	1179.2	68
7	30	7	10	6	2	0.17	0
8	45	4	7.5	40.5	3	1189.6	72
9	60	7	7.5	40.5	3	1262.3	71
10	30	7	7.5	40.5	3	1163.8	73
11	45	7	7.5	40.5	3	1022	69
12	30	4	10	75	2	172.4	61
13	30	10	5	6	4	5.11	0
14	45	10	7.5	40.5	3	1206.8	73

No.	DNA Template （ng/μL）	Cap Analog （mM）	T7 RNA Polymerase （U/μL）	Mg^{2+} （mM）	Reaction Time （h）	Yield （μg）	Integrity （%）
15	60	4	5	6	4	0.15	0
16	60	7	10	75	2	325.95	61
17	30	4	5	75	4	181.65	37
18	45	7	7.5	40.5	3	1221.3	72
19	60	10	5	75	2	284.6	64
20	30	10	5	75	2	73.8	64
21	60	4	10	6	2	0.52	0
22	45	7	7.5	75	3	273.6	52
23	60	10	5	75	4	280	44
24	30	4	5	6	2	0.16	0
25	60	10	5	6	2	0.02	0
26	45	7	5	40.5	3	1239.1	0.73
27	30	10	10	75	4	296.85	0.48
28	45	7	10	40.5	3	1233.5	0.72

Table S2. Encapsulation efficiency and Z-average Size and PDI of SaRBD vaccines with different integrity.

	0.37 V−Sa−RBD	0.64 V−Sa−RBD	0.80 V−Sa−RBD
Encapsulation efficiency（%）	94.6	95.2	94.8
Z-average Size（nm）	116.7	122.3	119.1
PDI	0.097	0.081	0.092
